# Spatial Engineering of Mammary Epithelial Cell Cultures with 3D Bioprinting Reveals Growth Control by Branch Point Proximity

**DOI:** 10.1007/s10911-024-09557-1

**Published:** 2024-02-28

**Authors:** Leena M. Koskinen, Lari Nieminen, Antti Arjonen, Camilo Guzmán, Markus Peurla, Emilia Peuhu

**Affiliations:** 1 Institute of Biomedicine, Cancer Laboratory FICAN West, University of Turku, Turku, Finland; 2 Turku Bioscience Centre, University of Turku and Åbo Akademi University, Turku, Finland; 3 Brinter Inc, Turku, Finland; 4 Euro-BioImaging ERIC, Turku, Finland

**Keywords:** 3D bioprinting, Branching, Mammary gland, Breast cancer, Ductal carcinoma in situ

## Abstract

The three-dimensional (3D) structure of the ductal epithelium and the surrounding extracellular matrix (ECM) are integral aspects of the breast tissue, and they have important roles during mammary gland development, function and malignancy. However, the architecture of the branched mammary epithelial network is poorly recapitulated in the current in vitro models. 3D bioprinting is an emerging approach to improve tissue-mimicry in cell culture. Here, we developed and optimized a protocol for 3D bioprinting of normal and cancerous mammary epithelial cells into a branched Y-shape to study the role of cell positioning in the regulation of cell proliferation and invasion. Non-cancerous cells formed continuous 3D cell networks with several organotypic features, whereas the ductal carcinoma in situ (DCIS) –like cancer cells exhibited aberrant basal polarization and defective formation of the basement membrane (BM). Quantitative analysis over time demonstrated that both normal and cancerous cells proliferate more at the branch tips compared to the trunk region of the 3D-bioprinted cultures, and particularly at the tip further away from the branch point. The location-specific rate of proliferation was independent of TGFβ signaling but invasion of the DCIS-like breast cancer cells was reduced upon the inhibition of TGFβ. Thus, our data demonstrate that the 3D-bioprinted cells can sense their position in the branched network of cells and proliferate at the tips, thus recapitulating this feature of mammary epithelial branching morphogenesis. In all, our results demonstrate the capacity of the developed 3D bioprinting method for quantitative analysis of the relationships between tissue structure and cell behavior in breast morphogenesis and cancer.

## Introduction

The mammary gland develops through branching morphogenesis when mammary epithelial ducts invade and branch out to form a tubular network through bifurcation of the terminal end buds (TEB) and lateral side-branching [1]. While the ductal outgrowth is induced by hormonal changes during puberty, further side-branching occurs in the adult gland during the reproductive cycles [2]. The entire ductal-lobular epithelial structure originating from the teat is surrounded by a basement membrane (BM), and embedded in connective and adipose tissue. The normal tissue organization and composition of the surrounding extracellular matrix (ECM) have been shown to regulate the development of the mammary epithelium, and to maintain tissue homeostasis [3–6]. However, the specific mechanisms that locally promote or inhibit cell proliferation, motility, and invasion in the growing tissue are not fully understood.

**Fig. 1:**
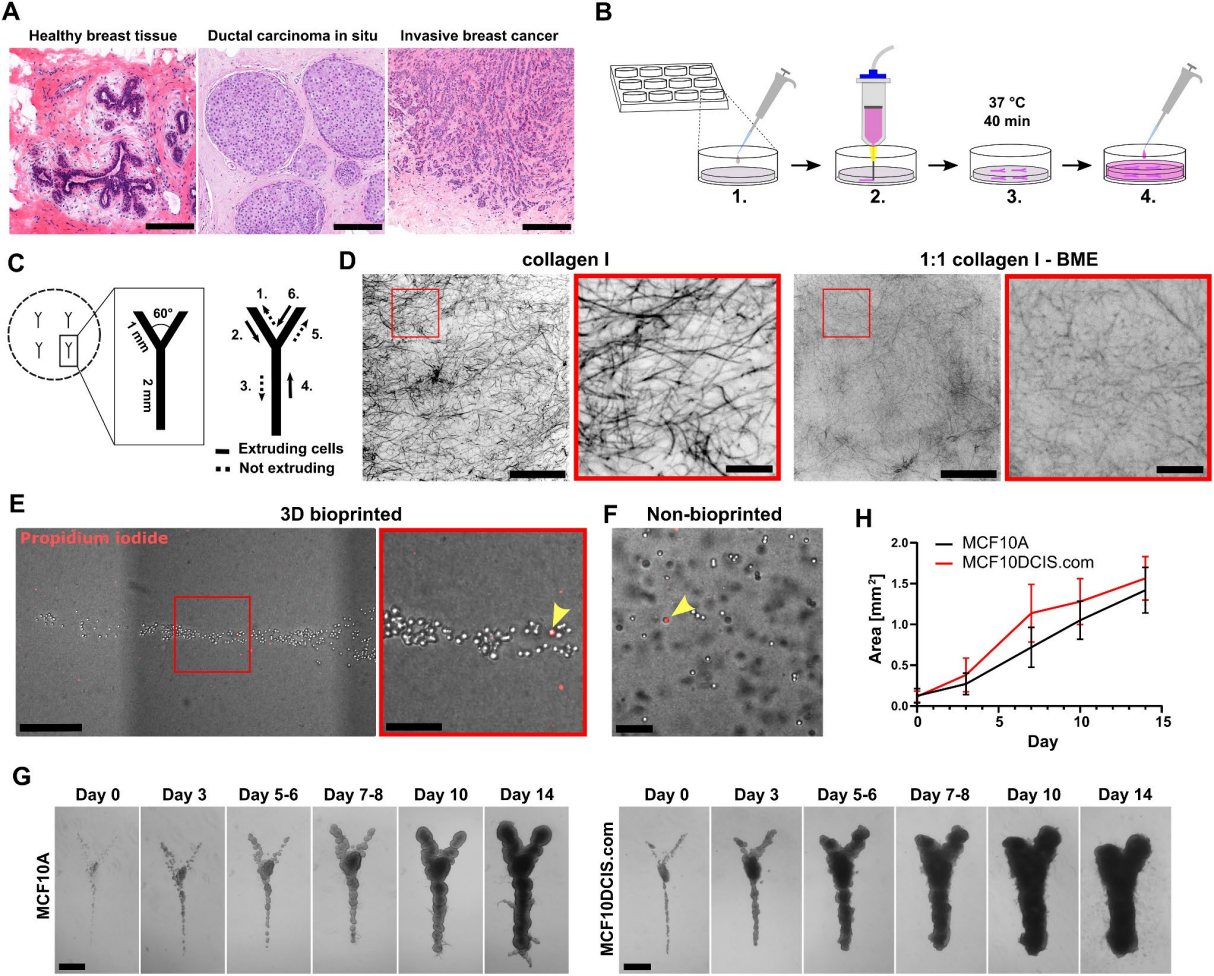
Extrusion 3D bioprinting of non-cancerous and cancerous breast epithelial cells. **A** Histological images of healthy human breast tissue, ductal carcinoma in situ (DCIS) and invasive breast cancer representing the gradual loss of mammary ductal architecture. **B** The workflow of 3D bioprinting. (1) Unpolymerized 1:1 collagen I:BME matrix is casted in the wells on a 12-well plate. (2) The cells are mixed in medium and printed inside the unpolymerized matrix. (3) The matrices are let to polymerize at 37 °C for 40 min. (4) Culture medium is added to the wells and the matrices are detached from the walls to allow floating. **C** The shape, dimensions and the sequence of 3D bioprinting the four Y-structures per well. **D** Collagen I fibers (CNA35-GFP, black) visualized by confocal microscopy (single z-planes) in the drop cast pure collagen I (left) and collagen I:BME gels (right). Magnified images are shown in the regions of interest (ROI). **E-F** The viability of 3D-bioprinted MCF10A cells **E** is high and comparable to cells that were mixed manually into the ECM gel **F** Propidium iodide labelled necrotic/late apoptotic cells (red) are indicated with yellow arrow heads. **G-H** Transmitted light microscopy imaging **G** and quantification of the growth area **H** of the 3D-bioprinted non-cancerous MCF10A and cancerous MCF10DCIS.com cell cultures on day 0–14. Mean ± SD, *n* = 21–31. Scale bars A: 200 μm; D: 50 μm, ROI 10 μm; E: 300 μm, ROI 100 μm; G: 500 μm

Various coordinated growth factor and ECM regulatory signals from the local stroma and the epithelium itself have been reported to guide mammary epithelial branching [7–10]. Yet, it is not known how the species-specific branching patterns are achieved in the mammary gland, and which homeostatic mechanisms limit excessive growth in the healthy mammary gland but are overcome in breast cancer. In ductal carcinoma in situ (DCIS), the malignant cells grow within the ductal lumen of the breast (Fig. 1A). While DCIS patients generally have a very good prognosis [11], the risk of invasive breast cancer is still increased [12], and DCIS is considered a precursor stage for invasive breast cancers. The progression of breast cancer to an invasive disease (Fig. 1A) shares some similarities with the tightly controlled developmental process of branching morphogenesis: they both involve active proliferation and motility of the cells towards the surrounding stromal tissue. Thus, understanding the control mechanisms of the normal morphogenesis could provide important insights of breast cancer.

The interactions between mammary epithelial cells and the ECM have traditionally been investigated by mixing the cells in a gel-forming matrix with the desired ECM components and casting the homogenous mix in wells or molds [13–15]. With this method, however, the spatial control of the distribution of cells or ECM components within the cultures is not possible, and recapitulation of the complex tissue organization in vivo is compromised. To improve tissue mimicry, multilayered three-dimensional (3D) cell cultures where the cells are seeded in cavities of desired shape have been developed [16–18]. However, these methods have limited versatility in terms of variation of the shape and components. Therefore, accurate and reproducible methods for creating more complex and spatially patterned 3D cell cultures have been lacking. 3D bioprinting has emerged as a novel method to position cells and ECM into desired geometries in 3D cell cultures in microscale resolution, and the 3D structures can be easily modified according to a digitally designed model [19]. Previously, larger continuous structures were produced by 3D bioprinting drops of normal mammary epithelial cells or breast cancer cells in the ECM at specific intervals, and allowing the formed organoids to gradually fuse [20–22]. However, no methods have previously been developed for 3D-bioprinted in vitro models combining a continuous cell network with a branched design.

In this study, we developed and optimized a protocol for 3D-bioprinted breast tissue cultures to study the effects of tissue geometry on the proliferation and invasion of normal mammary epithelial cells and of cells that form gradually progressing DCIS-like tumors in vivo [6, 23]. Our data demonstrate that the 3D-bioprinted mammary epithelial cell cultures retain a defined shape, and exhibit basal polarization and BM assembly. The cells grow according to their position in the Y-shaped design with the tips exhibiting a higher rate of proliferation, particularly further away from the branch point. The DCIS-like breast cancer cells also retain the printed shape and proliferate at the tips of the structure but fail to form a polarized basal epithelial layer, or a distinct BM, and invade preferably at the trunk region. The location-specific rate of proliferation was not regulated by TGFβ signaling while TGFβ promoted the invasion of DCIS-like breast cancer cells. In all, our results demonstrate the capacity of the developed method to address quantitatively potential relationships between the tissue structure and cell behavior in the context of breast morphogenesis and disease.

## Methods

### Human Breast Tissue

Healthy breast tissue was obtained from patients undergoing breast reduction mammoplasty, and breast cancer tissue was obtained from patients diagnosed with breast carcinoma. Tissues were voluntarily donated upon written informed consent at Turku University Hospital (Ethical approval ETKM 23/2018). The tissues were excised and examined by a clinical pathologist, processed to frozen tissue sections, and labelled by H&E staining by standard procedures.

### Cell Lines

The immortalized MCF10A breast epithelial cells, and their tumorigenic H-Ras transformed variant MCF10DCIS.com with stable expression of Lifeact-mRFP [24–26] were cultured in DMEM/F12 GlutaMAX™ (Gibco) supplemented with 5% horse serum (Gibco), 10 µg/ml insulin (Sigma), 5 µg/ml hydrocortisone (Sigma), 20 ng/ml epidermal growth factor (Sigma) and 100 ng/ml cholera toxin (Sigma) for a maximum of 30 passages.

### 3D Bioprinting

For 3D bioprinting, the cells were harvested with 0.25% trypsin-EDTA (Gibco) and resuspended in the culture medium to gain 3 × 10^6^ cells/ml cell suspension, which was used as such for 3D bioprinting. Collagen I (PureCol® EZ Gel, Advanced Biomatrix) and basement membrane extract (BME) (Cultrex® reduced growth factor BME, Trevigen) solution was prepared in 1:1 volume ratio to produce a matrix with 2.5 mg/ml collagen I and ~ 5 mg/ml BME for 3D bioprinting. All materials were kept chilled on ice until printing with Brinter® ONE 3D bioprinter (Brinter Inc.). Cold 1:1 collagen I:BME solution was pipetted into 12-well plate, 700–800 µl per well, right before printing, and the plate was placed in the 3D bioprinting chamber (room temperature, RT). Cell suspension was loaded into a printing syringe (Optimum® 10 cc, Nordson EFD) with a piston (Optimum® Blue LV Barrier, Nordson EFD) and a 100 μm needle tip nozzle (Optimum® general purpose tips, Nordson EFD). Four Y-shaped cell-laden structures were 3D-bioprinted per well inside the (unpolymerized) collagen-BME matrix. The structures were printed at 1 mm/s speed with 5 mbar pneumatic pressure. To solidify the matrix, the plate was moved to a 37 °C cell culture incubator for 40 min, after which warm culture medium was added to the wells, and the gels were detached from well edges. For viability assessment, propidium iodide (P3566, Invitrogen, 1:1000) was added into the medium of live 3D-bioprinted cultures, and imaged immediately. The 3D-bioprinted cultures were maintained for 14 days with medium replacement every 2–3 days, and finally fixed with 4% paraformaldehyde in PBS for 15 min.

### Clonal MCF10A Spheroids

The spheroids were grown from single MCF10A cells in gel droplets. For the gel matrix, collagen I and BME were mixed at ratios of 1:1 or 3:7 on ice. The droplets consisted of 30 µl of gel mixture and 15–30 cells added in 5 µl of culture medium. The droplets were pipetted on a pre-warmed 8-well µ-slide (Ibidi), the slide was turned upside down and kept at 37 °C for 30 min to solidify the droplets. Culture medium was added on top of the solidified gel droplets, and changed every 2–3 days until fixation with 4% paraformaldehyde in PBS on day 14.

### Inhibitor Studies

MCF10A and MCF10DCIS.com cells were plated on a 96 well plate, 16,000 and 12,000 cells per well, respectively. On the following day, DMSO or 1 µM A83-01 (Sigma) was added in culture medium as triplicates. The plate was imaged with IncuCyte® S3 live-cell analysis system (Sartorius) at 4-h intervals for 24 h. Confluency was calculated and normalized to 0-h timepoint in IncuCyte® program.

For preparation of western blot samples, MCF10A and MCF10DCIS.com cells were plated on a 12-well plate, 100,000 and 70,000 cells per well, respectively. On the following day, DMSO, 1 µM A83-01, 5 ng/ml TGFβ (PeproTech) or 1 µM A83-01 + 5 ng/ml TGFβ was added in culture medium. After 1-h incubation, the plate was placed on ice, the wells were washed with cold PBS, and the cells were scraped off and lysed with TX lysis buffer [50 mM Tris–HCl pH 7.5, 150 mM NaCl, 0.5% Triton X-100, 5% glycerol, 1% SDS, Complete protease inhibitor, PhosSTOP (Roche)] for western blotting.

After 3D bioprinting, DMSO or 1 µM A83-01 was added in the culture medium. The treatment was started on day 7 after printing, and continued for three days (until day 10), when the gels were fixed for further analysis.

### Western Blotting

The cell lysates were sonicated for 15 s after which 6x Laemmli sample buffer was added, and the lysates were incubated at 95 °C for 10 min. The lysates were loaded on 10-well 4–20% Mini-PROTEAN TGX gels (Bio-Rad) and run at 80 V for 20 min and then at 110 V for 1 h. The proteins were transferred on Trans-Blot Turbo Mini PVDF membranes (Bio-Rad) using Trans-Blot Turbo transfer system (Bio-Rad) and mixed MW program. The membranes were blocked in 5% BSA in 0.1% TBS-Tween for 1 h, at RT. The membranes were incubated overnight at 4 °C with primary antibodies against SMAD2/3 (D7G7, 8685 S, Cell Signaling, 1:500), pSMAD3 (C25A9, 9520 S, Cell Signaling, 1:500) or GAPDH (5G4cc, HyTest, 1:2000), followed by incubation in anti-rabbit (926-68073, IRDye, 1:4000) or anti-mouse (926-32212, IRDye, 1:4000) secondary antibodies for 1 h at RT. All antibodies were diluted in 5% BSA in 0.1% TBS-Tween, and the membranes were washed in 0.1% TBS-Tween for 3 × 5 min after each antibody incubation. The protein bands were detected with Odyssey CLx imaging system (LI-COR).

### Atomic-Force Microscopy Indentation

Atomic force microscopy (AFM) was performed on cell-free 1:1 collagen I:BME gels. The gels were made similarly as for 3D bioprinting and kept in culture medium at 37 °C for 14 days before AFM. All AFM indentations were performed using a JPK NanoWizard II AFM with its CellHesion module (JPK Instruments), mounted on a Carl Zeiss confocal microscope Zeiss LSM510 (Carl Zeiss AG). Triangular silicon nitride cantilevers with a spring constant of 0.06 N/m were custom fitted with borosilicate glass spheres 4.5 μm in diameter (Novascan Tech) and calibrated using the thermal noise method prior to each experiment [27]. The deflection sensitivity was determined in fluid using glass substrates as an infinitely stiff reference material. Gels were indented in cell culture medium at RT. A calibrated force of 4 nN was applied, and the Hertz model of impact [28] was used to determine the elastic properties of the tissue. The Young’s elastic modulus was calculated using the JPK data processing software (JPK DP version 4.2) and an input Poisson’s ratio of 0.5. Indentation was performed with gels coming from two independent preparations. For each gel, a total of 225 indentation curves distributed in three regions were performed, with a 5 × 5-point grid (100 × 100 µm^2^; 3 repeats per location) in each region. After determining that the data points showed a Gaussian distribution, the mean value and its standard deviation were calculated.

### Immunofluorescence Labelling and Imaging

The fixed 3D-bioprinted gels were permeabilized with 0.8% Triton-X100 (in PBS) for 1 h at RT, and incubated in blocking solution (10% horse serum (Gibco) in 0.1% TBS-Tween) for 2 h at RT or overnight at 4 °C. The gels were incubated with primary antibodies against integrin α6 (NKI-GoH3, MCA699GA, Bio-Rad, 1:800), laminin α5 (4C7, ab17107-1001, Abcam, 1:50), E-cadherin (24E10, 3195 S, Cell Signaling, 1:200), vimentin (V9, 347 M-1, Sigma, 1:1000) and Ki67 (GR3375556-1, ab15580, Abcam, 1:250) in the blocking solution for 3 days at 4 °C on a shaker. The gels were then washed 3 × 30 min with 0.1% TBS-Tween, followed by incubation with DAPI (Invitrogen, 1:2000), phalloidin atto-647 (Sigma, 1:700) and secondary antibodies (all 1:400) against mouse [Alexa Fluor 488 (A21202), Alexa Fluor 647 (A31571), Invitrogen], rat [Alexa Fluor 488 (A21208), Alexa Fluor 568 (A11077), Alexa Fluor 647 (A21247), Invitrogen] and rabbit [Alexa Fluor 488 (A21206), Alexa Fluor 568 (A10042), Alexa Fluor 647 (A31573), Invitrogen] in the blocking solution for 2 days at 4 °C on a shaker. The gels were washed 3 × 30 min with 0.1% TBS-Tween. For clearing, the gels were incubated in 80% glycerol overnight at 4 °C. Finally, the gels were washed with PBS and mounted on microscope slides with Mowiol® (Calbiochem) supplemented with 2.5% 1,4-diazabicyclo[2.2.2]octane (DABCO, Sigma-Aldrich).

The labelled gels were imaged with 3i Marianas CSU-W1 spinning disk confocal microscope (Intelligent Imaging Innovations, Inc.) equipped with Photometrics Prime BSI sCMOS camera (2048 × 2048 pixels, pixel size 6.5 × 6.5 μm). Bit depth of images was 16 bit and binning of 1 × 1 was used. The images were acquired with Zeiss Plan-Apochromat 10x/0.45 NA objective, Zeiss Plan-Apochromat 20x/0.8 NA objective, Zeiss LD Plan-Neofluar 20x/0.4 NA objective, Zeiss LD Plan-Neofluar 40x/0.6 NA objective, Zeiss C-Apochromat 40x/1.2NA water immersion objective and Zeiss Plan-Apochromat 63x/1.4 NA oil immersion objective. DAPI was excited with 405 nm solid state laser, and the emission light was collected with 445/45 nm filter. Alexa Fluor 488 was excited with 488 nm solid state laser, and the emission light was collected with 525/30 nm filter. Propidium iodide and Alexa Fluor 568 were excited with 561 nm solid-state laser, and the emission light was collected with 617/73 nm filter. Phalloidin atto-647 and Alexa Fluor 647 were excited with 640 nm solid state laser, and the emission light was collected with 692/40 nm filter. SlideBook 6 software was used in the image acquisition, and the images were analyzed with Fiji.

### Image Quantification

The outgrowth of the 3D-bioprinted cultures at different sub-regions was assessed by superimposing the transmitted light microscopy images from culture days 3 and 14, segmenting the composite image and dividing it into the sub-regions and measuring the relative change in culture area from day 3 to 14. The ratio of Ki67-positive nuclei was determined by dividing the maximum projection images of the cultures into the sub-regions, segmenting the DAPI and Ki67 signal areas and calculating their ratio. The area of invasion was determined by segmenting day 14 or day 10 transmitted light microscopy images of the cultures. The invasion areas were first erased from the segmented image by applying Fiji gray morphology function (radius: 30 (day 14 images) or 20 (day 10 images), type: circle, operator: open) to get the area of the main structure. Invasions not erased by the function were erased manually. The segmented area of the main structure was subtracted from the original segmented image to extract the invasion area, which was then divided into the sub-regions and calculated. The calculated invasion areas were divided by the average length of the respective sub-region outline to make the areas proportional to the size of the sub-region. Outline length was used instead of sub-region total area, because only the invasions beginning from and extending over the outlines of the main structure could be analysed.

### Statistical Analysis

Bar and line graphs were generated and statistical tests were performed using GraphPad Prism software. Normality of the data was tested with Shapiro-Wilk normality test. When two normally distributed groups were compared, t test (paired or unpaired according to the data, two-tailed) was used. When two non-normally distributed groups were compared, Wilcoxon matched-pairs signed rank test was used for paired data and Mann-Whitney test was used for unpaired data. P-values are designated in the graphs with ns (*p* > 0.05), * (*p* ≤ 0.05), ** (*p* ≤ 0.01), ***(*p* ≤ 0.001) and **** (*p* ≤ 0.0001). N-numbers for each graph are presented in figure legends. Data are presented as mean and standard deviation of the data.

### Code Availability

The G code for 3D bioprinting is available on Mendeley Data [29].

## Results

### Extrusion 3D Bioprinting of Non-Cancerous and Cancerous Mammary Epithelial Cells inside an ECM Gel

In order to model the ductal architecture of the mammary gland in vitro, we utilized extrusion 3D bioprinting. Our goal was to develop a 3D-bioprinted cell culture model, where the mammary epithelial cells would be positioned into a desired shape and fully embedded inside a 3D matrix that mimics the breast tissue ECM biochemically and mechanically. While the BM is rich in collagen IV and laminins [30, 31], collagen I is the predominant ECM component of the interstitial stroma [32, 33]. Therefore, gel forming biomolecules relevant to the breast tissue, including BM proteins and collagen I, were preferred in the selection of biomaterials for the matrix. Multiple biomaterial combinations were considered and tentatively tested. However, most of the materials formed solid scaffolds (alginate) or remained too fluid to maintain the printed shape (crosslinked hyaluronic acid) (data not shown). Of all tested materials, the combination of bovine collagen I and BME at 1:1 volume ratio (or 2.5 mg/ml and ~ 5 mg/ml, respectively) was considered as the most suitable candidate for the matrix due to its sufficient viscosity and gel-forming capacity. Hence, this combination was selected for further proof-of-principle studies (Fig. 1B).

To experimentally model the branched structure of the mammary epithelial ducts, we designed a simple Y-shape with a branch width of 100 μm (minimum nozzle size), and branch angle of 60˚ (Fig. 1C), roughly corresponding to the branching parameters observed in the human breast tissue [34]. The non-cancerous MCF10A breast epithelial cells and the breast carcinoma in situ -like MCF10DCIS.com cells were used for the 3D bioprinting to investigate the differences between normal and DCIS-stage growth patterns. As 3D bioprinting of cells suspended in the ECM gel resulted in poor shape retention (data not shown), the cells were 3D-bioprinted in medium using a needle nozzle directly inside the mixture of cold collagen I and BME, and the 3D matrix was let to polymerize at 37 °C. The polymerized matrices were then detached from the well walls to allow floating, which has been shown to promote mammary epithelial differentiation [10, 15], and the 3D-bioprinted cultures were grown up to 14 days (Fig. 1B). The average elastic modulus of the polymerized collagen I:BME matrix was 103.4 ± 14.3 Pa as measured by AFM indentation. In this matrix, collagen I was able to form fibers similar to pure bovine collagen I gels (Fig. 1D), which have previously been used for the culture of branching mammary epithelial organoids [15]. Cell viability remained high (> 95%) after 3D bioprinting (Fig. 1E) and was comparable to cells that were manually mixed into the collagen I:BME matrix (Fig. 1F). During the 14-day period, the 3D-bioprinted cultures grew gradually (Fig. 1H) and formed continuous multicellular structures maintaining the Y-shape (Fig. 1G). Although the cells remained mostly within the 3D-bioprinted shape, some invasion was observed (Fig. 1G). Overall, the method was accurate and reproducible.

### 3D-Bioprinted Breast Epithelial Cell Cultures Recapitulate Some Organotypic Features Including Basal Cell Polarization and BM Assembly

**Fig. 2:**
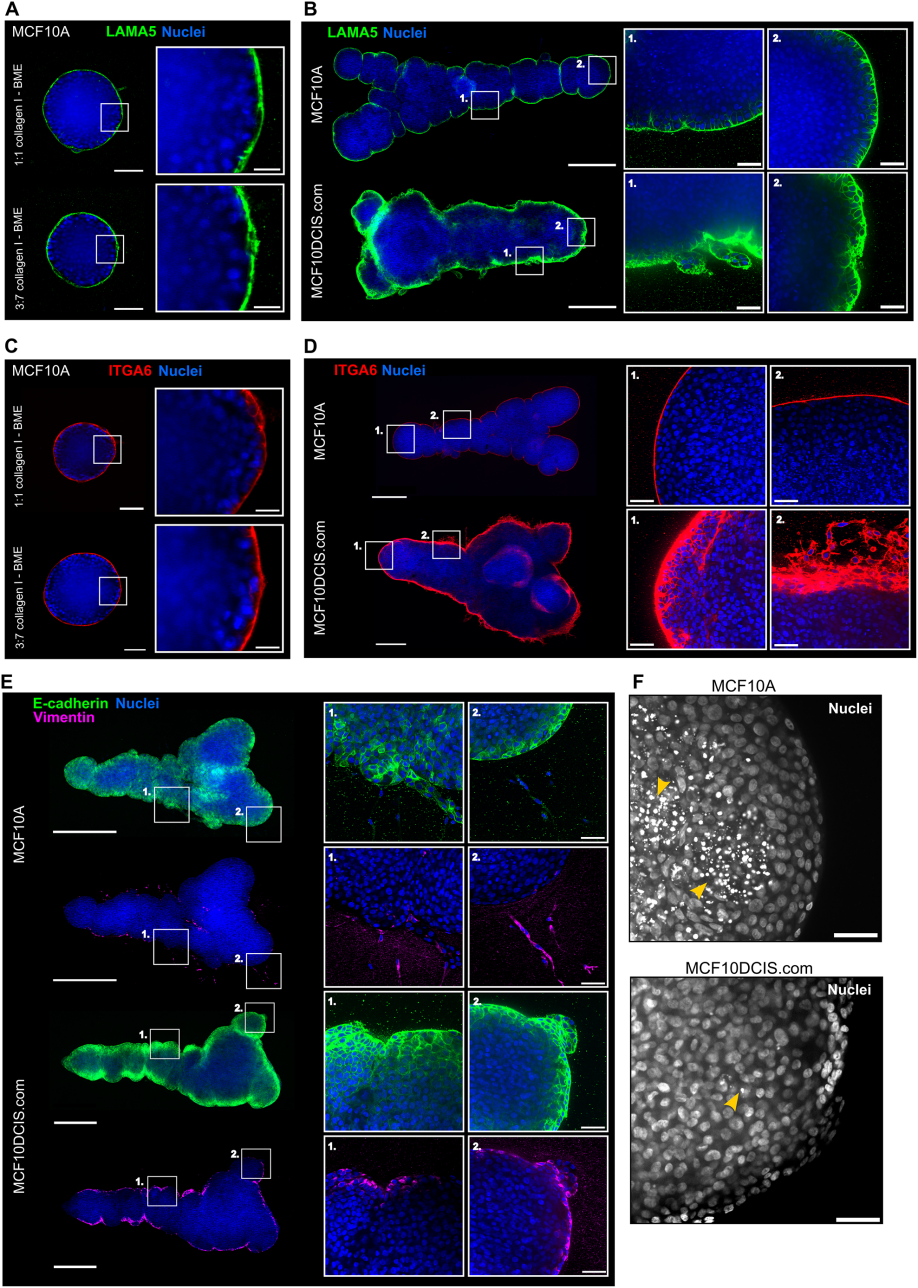
Extrusion 3D bioprinting of mammary epithelial cells inside an ECM leads to formation of polarized 3D cell cultures of a defined shape. **A-B** Immunofluorescence imaging of the BM protein laminin α5 (LAMA5, green) and nuclei (blue) in clonal spheroid cultures of MCF10A cells **A**, and 3D-bioprinted cultures of MCF10A and MCF10DCIS.com cells **B** on day 14. Magnified images are shown in the ROIs. The MCF10A spheroids embedded in an ECM with elevated BME content **A** produced results comparable to the ECM used in **B**. **C-D** Immunofluorescence imaging of the basal epithelial integrin α6 (ITGA6, green) and nuclei (blue) in clonal spheroid cultures of MCF10A cells **C**, and 3D-bioprinted cultures of MCF10A and MCF10DCIS.com cells **D** on day 14. Magnified images are shown in the ROIs. The MCF10A spheroids embedded in an ECM with elevated BME content **C** produced results comparable to the ECM used in **D**. **E** Immunofluorescence imaging of E-cadherin (green), vimentin (magenta) and nuclei (blue) in 3D-bioprinted cultures of MCF10A and MCF10DCIS.com cells on day 10. Magnified images are shown in the ROIs. **F** Immunofluorescence imaging of nuclei (DAPI) in the 3D-bioprinted cultures of MCF10A and MCF10DCIS.com cells on day 14. Some of the apoptotic cells with fragmented nuclei are indicated with yellow arrowheads. Scale bars A, C: 100 μm, ROI 25 μm; B, D, E: 500 μm, ROI 50 μm; F: 50 μm. Images represent the central plane of the cell cultures

Tissue-mimetic 3D environment is required for the formation of mammary gland -like structures in vitro [10, 15, 35]. When evaluating the ability of the conditions to support organotypic growth, features like BM assembly, basal epithelial polarization towards the BM, and lumen formation are often considered [36]. The 3D-bioprinted cultures demonstrated some organotypic features. Clonal MCF10A spheroids assemble a BM around them indicated by a layer of laminin α5 (LAMA5) at the cell-ECM interphase (Fig. 2A), and the 3D-bioprinted cultures formed a comparable BM structure (Fig. 2B). In the 3D-bioprinted MCF10DCIS.com cultures LAMA5 was also strongly expressed by the cells at the ECM boundary but the layer was much broader and less defined (Fig. 2B). The spheroid and 3D-bioprinted MCF10A cultures also showed basal polarization as the outermost layer of cells expressed the BM-binding integrin α6 towards the basal surface (Fig. 2C-D). Similar to the BM marker LAMA5, integrin α6 was expressed in a broad basal layer of MCF10DCIS.com cells. In line with poorer assembly of a BM barrier, invasive protrusions were commonly observed in the 3D-bioprinted MCF10CIS.com cultures (Fig. 2B, D).

Both cell lines expressed the epithelial marker E-cadherin in the 3D-bioprinted cultures (Fig. 2E). In the MCF10A cultures, the mesenchymal marker protein vimentin was detected in cells invading out of the Y-shape as well as in a few cells on the outermost cell layer of the structure. In contrast, most the cells in the outermost cell layer expressed vimentin in MCF10DCIS.com cultures (Fig. 2E). The inner cells of MCF10A spheroids have previously been shown to undergo apoptosis to form a hollow lumen in BME [37], or in mixed 1 mg/ml collagen I and BME matrix [38, 39], but not in mixed matrixes containing collagen I at 2.2 mg/ml concentration or higher [40]. Accordingly, a distinct lumen did not form in the 3D-bioprinted cell cultures in 2.5 mg/ml collagen I and BME (Fig. 2F). While many apoptotic nuclei could be detected inside the 3D-bioprinted MCF10A cultures, the MCF10DCIS.com cultures did not exhibit apoptosis induction (Fig. 2F), which is well aligned with the DCIS-like growth pattern of MCF10DCIS.com in vitro and in vivo [6].

### Proliferation and Outgrowth, but not Invasion, of Normal and DCIS-Like Mammary Epithelial Cells Occurs Predominantly at the Tips of the Y-Shaped Cultures

**Fig. 3:**
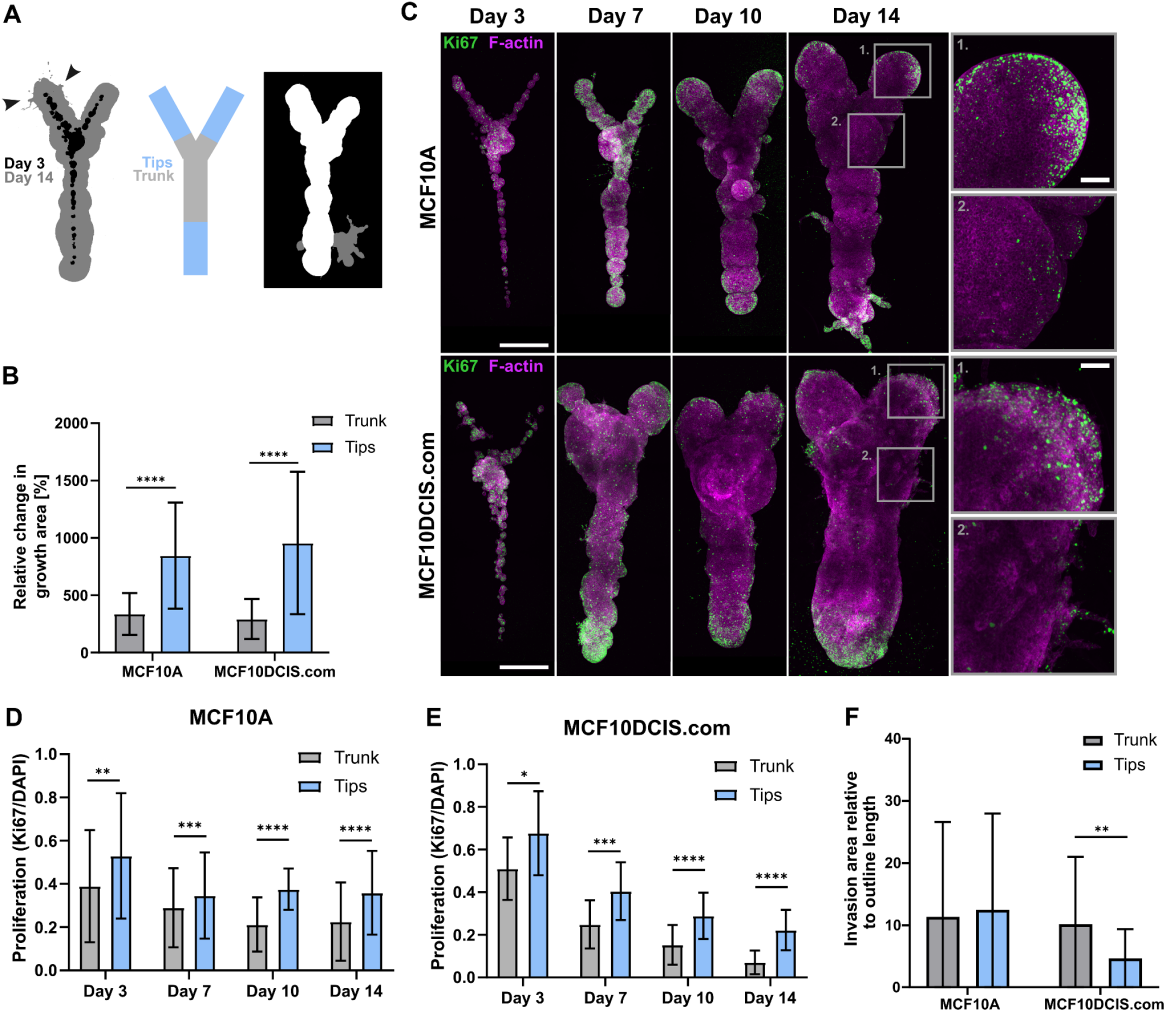
Proliferation and outgrowth, but not invasion, of normal and DCIS-like mammary epithelial cells occurs predominantly at the tips. **A** Segmentation of the growth area of a 3D-bioprinted cell culture on day 3 and day 14 (left), a schematic representation of the tip and trunk areas used for quantification of growth and proliferation (middle) and segmentation of the main structure growth area (white) and the invasion area (grey). **B** Quantification of the growth area expansion from day 3 to day 14 in the tips and the trunk of the 3D-bioprinted cell cultures using MCF10A or MCF10DCIS.com cells. Mean ± SD, *n* = 18–26, from 4 to 5 independent experiments, Wilcoxon matched-pairs signed rank test. **C** Immunofluorescence imaging of proliferating cells (Ki67, green) in 3D-bioprinted MCF10A and MCF10DCIS.com cell cultures on days 3–14. F-actin (magenta) was labelled with phalloidin. Magnified images are shown in ROIs. Images are maximum intensity projections. **D-E** Quantification of the amount of proliferation (Ki67-positive nuclear area) during days 3–14 in the tips and the trunk of the 3D-bioprinted MCF10A **D** and MCF10DCIS.com **E** cell cultures. Mean ± SD, **D** *n* = 10–11, 3–4 independent experiments per time point, **E** *n* = 5–12, 3–4 independent experiments per time point, paired t-test. **F** Quantification of the total invasion area in the tip or the trunk region per 3D-bioprinted structure relative to the length of the edge on day 14 using MCF10A or MCF10DCIS.com cells. Mean ± SD, *n* = 26–27, from 4 to 5 independent experiments, Wilcoxon matched-pairs signed rank test. Scale bars, **D**: 500 μm, inset 100 μm

During the monitoring of the growing 3D-bioprinted cultures, their tips were observed to gradually acquire a bulged shape (Fig. 1G, day 14). To quantify the local variation in growth, day 3 and day 14 light microscopy images were segmented and superimposed, and the culture area was divided into tip and trunk sub-regions (Fig. 3A). Indeed, the relative growth of the cultures was significantly larger at the tips than at the trunk region with both the non-cancerous MCF10A and the cancerous MCF10DCIS.com cells (Fig. 3B). To assess whether the higher outgrowth at the tip regions was due to localized increase in cell proliferation, the cultures were labelled for Ki67 proliferation marker. In general, the rate of proliferation was observed to gradually reduce during the culture (Fig. 3C-E). Interestingly, the Ki67-positive proliferating cells were enriched at the tip regions (Fig. 3C), and the fraction of Ki67-positive nuclei was significantly higher at the tip regions throughout the culture period with both cell lines (Fig. 3D-E), aligning well with the differences in sub-regional growth (Fig. 3B).

As previous data with microfabricated collagen I cavities indicated that invasion of mouse mammary epithelial cells occurs predominantly at tips of the cultures [16], we investigated the invasive behaviour of the non-cancerous breast epithelial and DCIS-like breast cancer cells in the 3D-bioprinted cultures. Invasive growth was defined as protrusions out of the main structure, and analysed by subtracting the main structure area from the invasion area and correlating it to the length of the region edge (Fig. 3A). Although the 3D-bioprinted structures grew and proliferated more at the tips compared to the trunk region in both normal and breast cancer cell cultures (Fig. 3B-E), the area of cell invasion did not differ between the tip and trunk regions of the MCF10A cells (Fig. 3F). Interestingly, the MCF10DCIS.com breast cancer cultures exhibited more invasion in the trunk region compared to the tips (Fig. 3F). In all, our data suggest that proliferation and invasion of breast cancer cells may preferably occur at distinct sites with different structural geometry.

### The Proximity of a Branch Point Inhibits Cell Proliferation in 3D-Bioprinted Cell Cultures

**Fig. 4:**
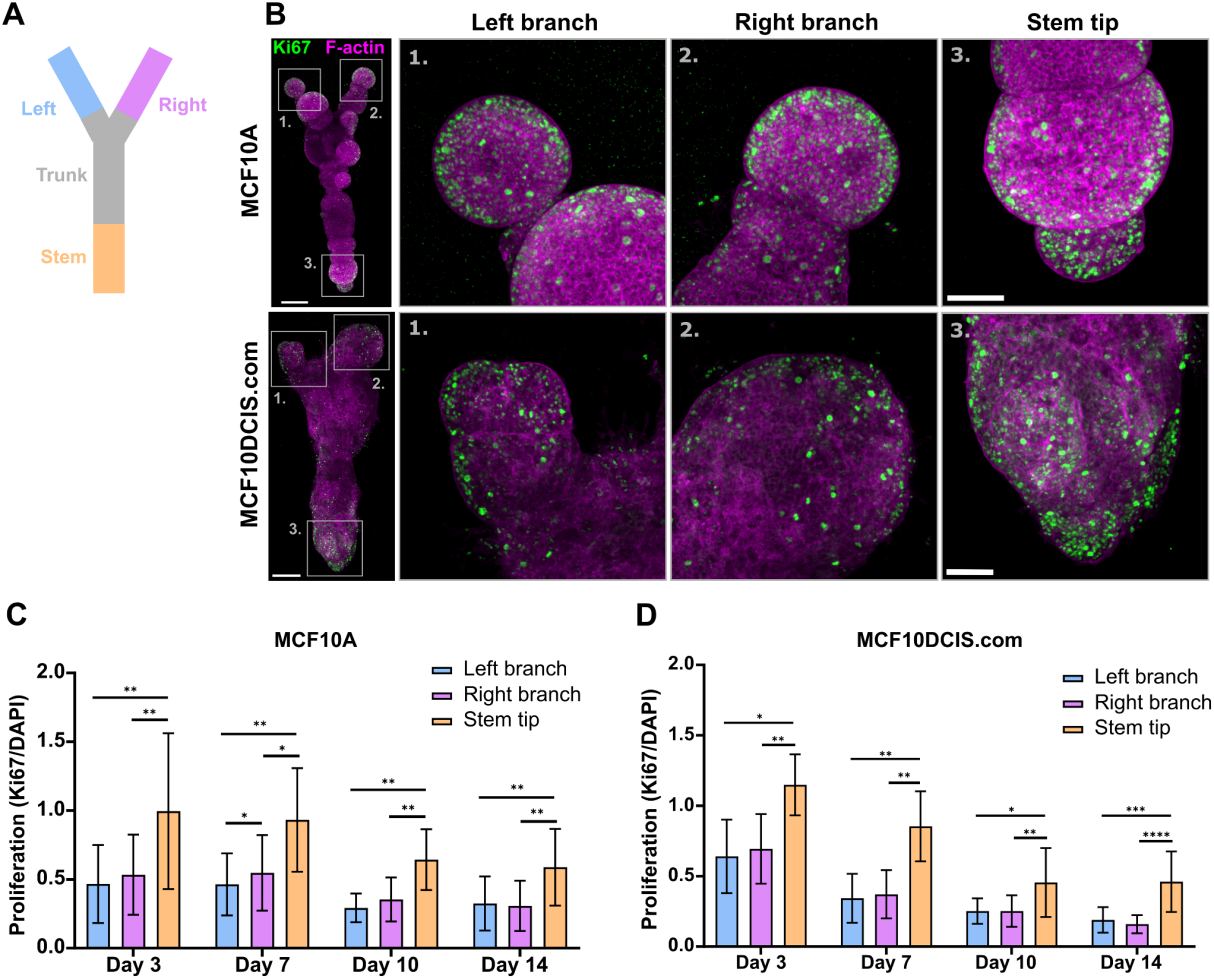
The proximity of a branch point inhibits proliferation of normal and DCIS-like mammary epithelial cells. **A** A schematic representation of the tip areas used for quantification of proliferation. **B** Immunofluorescence imaging of proliferating cells (Ki67, green) in 3D-bioprinted MCF10A and MCF10DCIS.com cell cultures on day 10. F-actin (magenta) was labelled with phalloidin. Magnified images are shown in ROIs. Images are maximum intensity projections. **C-D** Quantification of the amount of proliferation (Ki67-positive nuclear area) during days 3–14 in the different tip areas of the 3D-bioprinted MCF10A **C** and MCF10DCIS.com **D** cell cultures. Mean ± SD, **C** *n* = 10–11, 3–4 independent experiments per time point **D** *n* = 5–12, 3–4 independent experiments per time point, paired t-test. Scale bars, A: 300 μm, inset 100 μm

In the mammary gland, the vicinity of a branch point inhibits the formation of a new branch thus regulating the formation of a branched network with organotypic characteristics [41]. To evaluate the capacity of the 3D-bioprinted cell cultures with a Y-shape to model this behaviour, the rate of proliferation was compared between the different tip regions by analysis of the proportion of Ki67-positive nuclei on day 3–14 post 3D bioprinting (Fig. 4A-B). Indeed, proliferation was significantly higher at the stem tip, further away from the branch point compared to the left and right branch with both cell lines throughout the two-week culture period (Fig. 4C-D). Thus, despite their seemingly comparable size in the beginning of the cultures (Figs. 1G and 3C), the different tip regions exhibit differential capacities for proliferation depending on their location within the 3D-bioprinted structure.

### Inhibition of TGFβ Signalling does not Disrupt the Spatial Control of Cell Proliferation

**Fig. 5:**
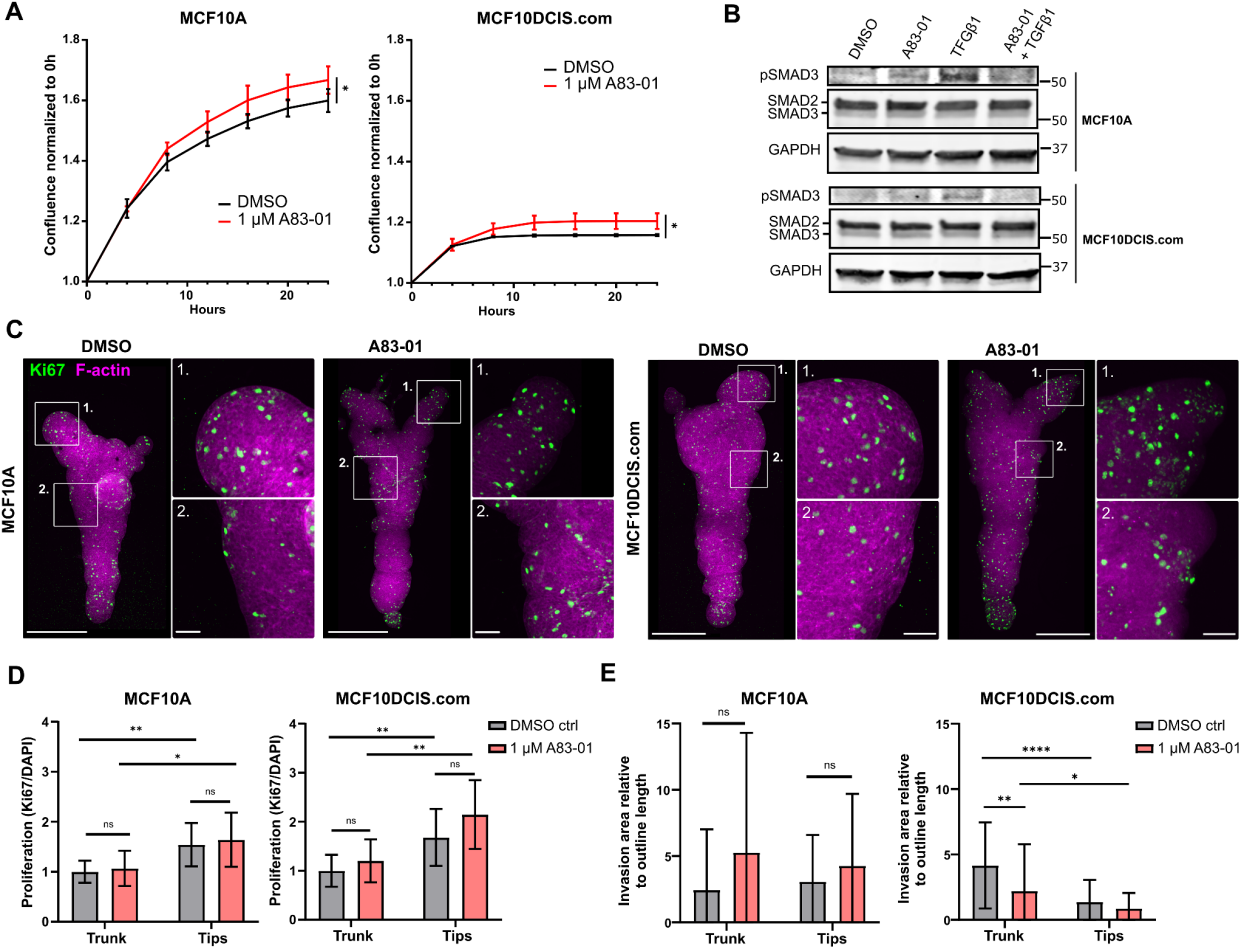
Inhibition of TGFβ signalling does not disrupt the spatial control of cell proliferation but reduces the invasion DCIS-like breast cancer cells. **A** The confluence of MCF10A (left, paired t-test) and MCF10DCIS.com (right, Wilcoxon matched-pairs signed rank test) cells treated with DMSO or 1 µM TGFβ type I receptor inhibitor A83-01 for 24 h. Mean ± SD, *n* = 2–3 wells per condition, one experiment. **B** Western blot analysis of SMAD3 phosphorylation (pSMAD3) in MCF10A and MCF10DCIS.com cells treated with DMSO, 1 µM A83-01, 5 ng/ml TGFβ or 1 µM A83-01 + 5 ng/ml TGFβ1 for 1 h. The total level of SMAD2/3 was blotted for reference and GAPDH served as a control for protein loading. **C** Immunofluorescence imaging of proliferating cells (Ki67, green) on day 10 in 3D-bioprinted MCF10A and MCF10DCIS.com cell cultures treated with DMSO or A83-01 for 3 days. F-actin (magenta) was labelled with phalloidin. Magnified images are shown in ROIs. Images are maximum intensity projections. **D** Quantification of the amount of proliferation (Ki67-positive nuclear area) on day 10 at the tips and the trunk of the 3D-bioprinted MCF10A (left) and MCF10DCIS.com (right) cell cultures treated with DMSO or A83-01 for 3 days. Data are normalized to the trunk region of the DMSO control. Mean ± SD, *n* = 8–11, 2–3 independent experiments, unpaired t-test. **E** Quantification of the total invasive area relative to the average length of tip or trunk outline on day 10 in the 3D-bioprinted MCF10A (left) and MCF10DCIS.com (right) cell cultures treated with DMSO or A83-01 for 3 days. Mean ± SD, *n* = 4–19, 3 independent experiments, Mann-Whitney test (DMSO vs. A83-01) and Wilcoxon matched-pairs signed rank test (tips vs. trunk). Scale bars, C: 500 μm, ROI 100 μm

Based on previous studies demonstrating that the secretion of TGFβ from normal mammary epithelial cells inhibits the invasion of the cells at the trunk of tube-shaped cell cultures in collagen I cavities [16], we hypothesized that localized secretion of an inhibitory molecule, such as TGFβ, from the branch region could regulate the behavior of cells in its proximity. To test this hypothesis, the 3D-bioprinted cultures were treated with A83-01, a small-molecule inhibitor for TGFβ type I receptor (TGFβRI) at a commonly used concentration [42] that increased the in vitro growth of MCF10A and MCF10DCIS.com cells (Fig. 5A) as expected based on the growth inhibitory effect of TGFβ on most epithelial cells [43]. The inhibition of TGFβ signalling by A83-01 was further validated by assessing SMAD phosphorylation downstream of TGFβRI activation. The phosphorylation of SMAD3 was increased by a treatment with recombinant TGFβ1, as expected, and the inhibitor of TGFβRI abolished this effect (Fig. 5B). The 3D-bioprinted cultures were treated with 1 µM A83-01 on days 7–10, which is a time period when a continuous structure had already been formed from single cells and the rate of proliferation was still high (Figs. 1G-H and 3C-E). The effect of the treatment on proliferation and invasion was evaluated at day 10 (Fig. 5C-E). Our data demonstrate that the inhibition of TGFβRI did not disrupt the preferential cell proliferation at tips compared to the trunk region (Fig. 5C-D), suggesting that regulatory mechanisms other than TGFβ signaling inhibit the proliferation at the trunk region, including the branch point.

At this earlier time point (day 10), a lower amount of cell invasion could be observed (Fig. 5E) compared to day 14 (Fig. 3F). As on day 14 (Fig. 3F), MCF10DCIS.com cells exhibited a higher rate of invasion at the trunk region compared to the tips (Fig. 5E). Upon treatment with A83-01 this difference was reduced as the invasion of the MCF10DCIS.com cells in the trunk region was decreased compared to control cells (Fig. 5E). In MCF10A cultures, the invasion occurred equally, and with high variability, at all regions, and the inhibition of TGFβ signaling did not have a significant effect, although a subtle increasing trend in invasion could be observed (Fig. 5E). These data suggest that TGFβ promotes the preferential invasion of breast cancer cells at the trunk region whereas cell proliferation occurs predominantly at the tips independent of TGFβ signalling.

## Discussion

Efficient investigation of the relationships between tissue structure and cell behavior in multicellular 3D systems requires experimental setups that allow flexible engineering of the shape and size of the structure, and quantitative analysis of measurable readouts. Compared to collagen I stamping [16] or micropatterning [44], which have previously been utilised to this end, 3D bioprinting offers easier modification of the structural design and enables the controlled incorporation of multiple components such as ECM molecules or different cell types. Previous studies using 3D bioprinting in the field of mammary gland biology have focused on creating improved co-culture and drug screening systems for breast cancer [45–50]. In a few studies, mammary epithelial cells have been mixed in a hydrogel and 3D-bioprinted as simple layers, lines, cylinders or grids that do not recapitulate the branched epithelial structure [48, 51–53]. Larger continuous organoids were previously generated by 3D bioprinting droplets of mammary epithelial cells inside a polymerized collagen I matrix, that over time formed organoids and fused into one continuous structure [22]. However, our study provides the first in vitro model for direct 3D bioprinting of mammary epithelial cells into a continuous cell network with a branched design. Importantly, it does not compromise cell viability or require potentially damaging chemical or UV-mediated crosslinking methods often employed in 3D bioprinting [54, 55].

The results of our study demonstrate that non-cancerous breast epithelial cells form continuous and polarized structures after 3D bioprinting. In turn, the DCIS-like cancer cells lack clear basal epithelial polarization or a defined BM layer, as expected based on their tumorigenic phenotype [6, 23]. Neither cell line produced cultures with lumen in the 1:1 matrix with 2.5 mg/ml collagen I, which has also previously shown to prevent lumen formation [40]. Although sufficiently high collagen I concentration is required for the detachment and floating of the gels, the matrix could potentially be optimized further to promote lumen and epithelial bilayer formation. Our data also suggest that both of the tested cell types are able to respond to their location within the Y-shaped cultures by elevated cell proliferation at the tips of the shape, particularly more distal to the Y branch point. This implies that the branch point or the branches themselves are providing one or multiple graded cues for the regulation of cell cycle activity at the tips. However, the exact nature of the cues remains unknown. Although the developed method does not recapitulate the hollow lumen or the bilayered epithelium of mammary ducts, nor presents a biologically accurate model of the branch point, it possesses the ability to unveil patterns in cell behavior associated with tissue geometry, which can be further explored through in vivo or ex vivo methods.

The links between tissue geometry and cell proliferation or invasion have previously been studied with mouse mammary epithelial cells seeded in cavities of collagen I where the cells lined the walls of the cavities and formed epithelial cell tubules. Homogenous proliferation was observed in these tubules but cell invasion occurred preferably at the tips, where TGFβ signalling was lower than in the trunk where TGFβ secretion inhibited cell invasion [16]. In our 3D-bioprinted non-cancerous human breast epithelial cell cultures, proliferation occurred preferably at the tips but cell invasion occurred variably both at the tips and the trunk regions. The inhibition of TGFβ signaling in these cells only had a slightly increasing, but statistically not significant effect on the invasion and no effect on cell proliferation in the 3D-bioprinted cultures. In embryonic mouse mammary epithelial explants, the tip bifurcation or branch elongation have been shown to depend more on cell motility than proliferation [56]. In all, these data imply that cell motility, and its regulation by TGFβ, could be particularly important for the invasive branch elongation process, whereas the tip-specific proliferation appears to be regulated through other pathways. The higher rate of proliferation at the tip regions of the 3D-bioprinted cell cultures could, for example, be related to the more curved form of the tips as increased curvature has been shown to promote the proliferation of cells [57].

In breast cancer, the tissue structure is gradually lost due to the increasing mammary ductal diameter, disrupted BM integrity, changing stromal ECM composition and tissue stiffness, and reduced proportion of adipose tissue [58–61]. Whether or not the invasive changes occur equally within the mammary ductal network or predominantly at distinct ‘weak’ points of the ductal geometry has not been fully elucidated. The geometry of the epithelium has previously been suggested to regulate breast cancer cell behavior with the regions of higher mechanical stress, such as the ends of the collagen I cavities, being more permissive to proliferation of non-invasive breast cancer cells, while invasive breast cancer cells were not affected by the location [62]. Our data with DCIS-like breast cancer cells in mixed BME and collagen I environment also revealed more prominent proliferation at the tips of the Y-shape. Interestingly, higher degree of invasion was detected at the trunk region of the 3D-bioprinted Y-shape, an effect reduced by TGFβRI inhibitor. The MCF10DCIS.com cell cultures had higher levels of vimentin than non-cancerous cultures indicative of a more mesenchymal phenotype. TGFβ has been shown to promote epithelial-to-mesenchymal transition (EMT) in mammary epithelial cells [63], and therefore, it is plausible that inhibiting TGFβ signaling reduces EMT, and the invasive traits associated with it, in DCIS-like cancer cell cultures.

In addition to the potential regulatory role of the ductal geometry, cell adhesion to the ECM guides mammary gland development [5]. BME has been shown to inhibit the formation of branched or tubular structures in a dose-dependent manner [64], and, instead, to promote the formation of alveolar structures [65]. In contrast, collagen I matrix mimics the stroma of the mammary gland, and favors branch formation and elongation in vitro [15, 35]. The source of collagen I affects the biomechanical properties of the 3D hydrogel: Rat-tail collagen gel exhibits a denser fiber network with less prominent fibers compared to bovine collagen [66] which is pepsin-digested and has slower gelation time than rat-tail collagen. The elastic modulus of bovine collagen was previously measured to be two-fold lower than of rat-tail collagen at 1.7 mg/ml (28 vs. 51 Pa) [66]. Accordingly, our gel containing 2.5 mg/ml bovine collagen had a stiffness of 103 Pa, representing the lower range of previously reported normal human mammary gland stiffness (100 Pa–1 kPa) [67]. Both rat tail collagen [21] and bovine collagen [51, 53] have been used in 3D bioprinting. As the collagen I hydrogels of different origins are different in terms of fibril diameter, fiber density, and network stiffness, it is likely to have important implications on how the cells respond to the matrices and the functionality of the hydrogel matrices 3D bioprinting applications.

Studies on mouse mammary glands have also revealed that different ECM components are deposited at distinct areas during branching morphogenesis: Collagen I is deposited especially around the established ducts, and laminin and hyaluronic acid are more abundant at the TEBs [68, 69]. The spatial patterning of the ECM suggests that ECM molecules have distinct roles in regulation of mammary gland development and homeostasis. Moreover, the patterning of ECM may provide favorable sites for cancer progression, while the malignant development itself is associated with further changes in the ECM, including increased expression [70, 71], crosslinking [72] and remodelling of ECM molecules, which have been shown to promote many aspects of breast cancer progression and therapy resistance. 3D bioprinting offers interesting opportunities for further assessment of the interplay between the ECM patterns and tumor geometry, an aspect that is particularly relevant for the early stages of breast cancer progression from in situ to invasive disease. Through incorporation of additional bio inks, our 3D bioprinting method will also enable the investigation of the specific roles of the ECM biomolecules at specific locations of the structure in non-cancerous and cancerous breast epithelial cell cultures. Uncovering the mechanisms by which tissue geometry and the ECM control normal mammary gland growth and homeostasis, as well as invasive progression of breast cancer, may reveal novel regulatory mechanisms that could be exploited in diagnostics and therapeutic targeting of breast cancer.

## Data Availability

No datasets were generated or analysed during the current study.
